# How to Perform Cardiac Contrast-Enhanced Ultrasound (cCEUS): Part I

**DOI:** 10.3390/diagnostics15141743

**Published:** 2025-07-09

**Authors:** Harald Becher, Andreas Helfen, Guido Michels, Nicola Gaibazzi, Roxy Senior, Christoph Frank Dietrich

**Affiliations:** 1 Mazankowski Alberta Heart Institute, Edmonton, AB T6G 2B7, Canada; harald@ualberta.ca; 2 St. Marien Hospital, 44534 Lünen, Germany; a.helfen@t-online.de; 3 Notfallzentrum, Krankenhaus der Barmherzigen Brüder Trier, 54292 Trier, Germany; g.michels@bbtgruppe.de; 4 Department of Cardiology, University of Parma, 43124 Parma, Italy; ngaibazzi@gmail.com; 5 Royal Brompton Hospital, National Heart and Lung Institute, Imperial College, London SW3 6NP, UK; r.senior@imperial.ac.uk; 6 Department General Internal Medicine (DAIM), Hospitals Hirslanden Bern Beau Site, Salem and Permanence, 3013 Bern, Switzerland

**Keywords:** echocardiography, contrast-enhanced ultrasound, ultrasound enhancing agent, contrast agent, LV function, cardiomyopathy, thrombus, tumor

## Abstract

Ultrasound enhancing agents (UEAs, formerly called contrast agents) for assessments of the left heart have improved the applicability of echocardiography and the accuracy of echocardiographic measurements. UEAs have been recommended for several diagnostic echocardiographic procedures by national and supernational agencies. The increased use of UEAs during the last years provided more evidence and experience in clinical practice data which is helpful for optimizing the UEA procedures and which will be useful for both newcomers to UEA in echocardiography and sonographers/physicians with experience in echocardiography with UEAs. In two parts, this review focuses on the “how to do” for the approved UEA applications. This is part 1, covering the available UEAs and providing specific guidance on the assessment of global and regional LV function. Part 2 covers the imaging of myocardial disease and masses as well as myocardial perfusion. Recommendations include the application of UEAs in two-dimensional echocardiography as there is limited data on three-dimensional echocardiography. A step-by-step approach is proposed for each of the procedures as well as guidance on how to interpret recordings and how to report them.

## 1. Introduction

Despite major advances in echocardiographic technology, echocardiographers still struggle with suboptimal recordings and limited accuracy of measurements in many patients. Contrast-enhanced ultrasound (CEUS) has mitigated the limitations of conventional echocardiography and has become an indispensable tool for state-of-the-art echocardiography laboratories [1].

The growing use of CEUS and recent studies have provided fresh insights regarding the application, dosage, quality control, and interpretation of echocardiography with UEAs, as well as clinically relevant aspects which have not been or have been incompletely stated in the previous literature. There is also more experience with pitfalls and troubleshooting. In this article, we integrate the new information with previously published recommendations to create an up-to-date and comprehensive paper on how to perform contrast-enhanced 2D echocardiography in clinical practice.

To standardize CEUS techniques, experts created a discussion forum to standardize the technique for contrast-enhanced echocardiography according to the published evidence and available guidelines. The World Federation for Ultrasound in Medicine and Biology (WFUMB) created a discussion forum to standardize the technique for CEUS examination of applications for contrast-enhanced ultrasound (CEUS) [2,3,4,5,6,7]. Ultrasound enhancing agents (UEAs) have been licensed for left ventricular cavity opacification, resulting in a better delineation of the endocardial border, and are increasingly used in echocardiography laboratories [8,9,10]. International and national societies for cardiovascular imaging have published indications and some guidance on how to perform echocardiography with UEAs [11,12,13,14,15,16]. However, more specific advice is rare and dated; the only compendium for clinical practice was published in 2019 [17].

This article provides updated and specific advice on the use of UEAs for clinical decision-making in echocardiography. The imaging parameters and dosage of UEAs for optimal recordings are included as well as specific guidance on how to analyze contrast-enhanced studies and report the findings. Advancements of echocardiographic technology and analysis tools are included which are likely to become clinically available within the coming five years.

This is a two-part article with part 1 covering the available UEAs and imaging methods and providing specific guidance on the assessment of global and regional LV function. The focus of part 2 is on the imaging of myocardial disease and masses, as well as myocardial perfusion.

### 1.1. Brief History of Cardiac CEUS

The development of contrast echocardiography started 50 years ago [8,9,10]. The best contrast effects were obtained when suspensions of gas-filled microbubbles were injected.

For a long time, mainly agitated saline was available for intravenous injection, which could not cross the pulmonary circulation. This has limited its use to enhanced visualization of the right heart and the diagnosis of intracardiac and intrapulmonary shunts. For the assessment of left-sided heart disease, more stable UEAs with microbubbles, but also specific imaging techniques, had to be developed. After 1990, several intravenously injectable UEAs were approved. Imaging was enhanced by Harmonic Imaging and contrast-specific ultrasound methods, which became available on echocardiography devices. Several major clinical trials were performed to evaluate the effectiveness of contrast echocardiography for the assessment of LV function, myocardial disease, and cardiac masses [18,19,20,21]. The first guidelines for the use of UEAs were published by the American Society of Echocardiography in 2008, and the last update was published in 2018 [15]. The most recent updates of practical guidelines were published by the British Society of Echocardiography in 2023 and by the International Contrast Ultrasound Society (ICUS) in 2022 [11,14].

### 1.2. Specific Terms for Cardiac Contrast-Enhanced Ultrasound

The American Society of Echocardiography introduced the term **ultrasound enhancing agent (UEA)** to be used instead of ultrasound contrast agent [13,14,15]. This has been helpful when explaining the procedure to patients for echocardiography with contrast agents. These patients often have some knowledge of worsening renal function when X-ray or older MRI contrast agents are administered, which is not true for UEAs. The approved UEAs for echocardiography can be administered even in patients with end-stage renal disease [22,23,24,25]. Using the term UEA instead of a “contrast” agent helps to clarify the difference between ultrasound and X-ray contrast agents and to reduce patient anxiety. In echocardiography, the distance between the transducer and the blood pool is smaller compared to abdominal ultrasound. In apical windows, which are most frequently used for recordings with UEAs, the apical RV and LV cavities are in the nearfield of the transducer, which potentially exposes the microbubbles to higher acoustic powers, resulting in the destruction of the microbubbles and a reduced contrast effect. **Contrast-specific imaging modalities** are required to reduce microbubble loss and provide adequate recordings [11,13,14,15,16,17]. The metric for the transmitted ultrasound power is the **mechanical index (MI)**—a dimensionless number that sonographers and physicians should know about and be able to adjust. In non-contrast 2D echocardiography, the MI is usually >0.8. However, for most UEA studies, it is below 0.2 (a low-MI setting). At this level, destruction of the microbubbles is minimized, and intense opacification of the blood in the cardiac cavities is found after intravenous injections of UEAs. Microbubbles in the myocardial blood pool can be displayed and quantified, which allows the assessment of myocardial perfusion in real time; Doppler epicardial coronary artery flow is also enhanced, especially in the left anterior descending artery, making Doppler coronary flow reserve measurement routinely feasible [26].

While scanning in the low-MI setting (<0.2), a **flash** can be applied to clear microbubbles. A flash button is available on most echocardiography scanners with contrast-specific imaging modalities. By pushing the flash button, the MI goes up to a value used for non-contrast echocardiography for a predefined number of frames [17]. This action can also be helpful in reducing signals from the microbubbles by reducing the concentration when the opacification of the blood pool and myocardium is too intense. The replenishment of the UEA in the myocardium after a flash has been used to assess myocardial perfusion (see part 2).

**Triggered imaging** has been used to enhance myocardial contrast signals for myocardial perfusion imaging. Instead of continuous transmission, the ultrasound is intermittently paused. The ultrasound transmission is turned on only for the duration of one frame—usually triggered by the R wave of the ECG. Even with a low-MI contrast-specific method, there is some destruction of microbubbles in the ultrasound field. When the microbubbles are not exposed to ultrasound, more microbubbles can fill the myocardial vessels compared to continuous imaging. However, simultaneous assessments of wall motion are not possible with triggered imaging. The most advanced contrast-specific imaging modalities usually provide adequate myocardial signals for myocardial perfusion imaging as well as in real-time imaging, with the advantage of simultaneously displaying wall motion and perfusion.

## 2. Ultrasound Enhancing Agents (UEAs), Doses, and Dynamics

Three UEAs have been approved for echocardiography [Table 1].

UEAs consist of microbubbles, which are small and durable enough to cross the pulmonary microcirculation and reach the arterial system following intravenous injections. Stability is achieved by the combination of a thin shell and fluorinated gas inside the shell. The differences between the three approved UEAs relate to different gases and compositions of the shells [Table 1]. Despite these differences, all three commercially available contrast agents are suitable for the assessment of LV function, structural LV abnormalities, masses, and myocardial perfusion. UEAs can be administered as a bolus or an infusion [Table 2].

After intravenous injection, the microbubbles of the UEAs remain intravascular. The acoustic pressure imparted by the transducer in the heart chambers and shear forces in the vessels lead to the destruction of some of the microbubbles. Even with very-low-MI contrast-specific imaging, microbubbles dissipate as the thin shells break within minutes when the microbubbles are exposed to ultrasound. At this stage, the contrast effect is gone. The fragments of the shell are metabolized in the liver, and the gas is finally exhaled through the lungs. UEAs can be administered to patients with end-stage kidney disease. Depending on the dosage and the power of the transmitted ultrasound, it takes up to 10 and 30 min until no more contrast signals are detected by echocardiography.

The dosages in package inserts of the UEAs have been derived from studies that were performed to obtain approval of the UEAs many years ago. In the meantime, more sensitive contrast-specific imaging modalities have become available, and there is more experience with diluting UEAs. The dosages and recommended dilutions listed in Table 2 consider these developments for bolus injections and infusions. Constant infusions of UEAs are suggested for the assessment of myocardial perfusion, but slow bolus injections have also been reported (SonoVue^®^ 0.4 or 0.3 mL followed by 5 mL of normal saline over 20 s, or 0.2 mL Luminity/Definity^®^ diluted with 1 mL normal saline over 20 s) [27]. Using the dosages of the package inserts on state-of-the-art echo machines can lead to image artifacts, mainly related to attenuation, affecting the visualization of structures at greater depths.

### 2.1. UEA Contraindications and Pre-Procedure Assessment

Recent data from Strom et al. showed that serious adverse events were uncommon in a large US nationwide claims analysis [28]. Critical cardiopulmonary reactions and fatalities are uncommon during or after UEA administration [29,30,31,32,33]. Low-concentration applications of the UEA Definity^®^ minimize back pain and can be performed without a flush [34]. However, all patients should be monitored for cardio-respiratory instability after UEA injection, especially those with acute coronary syndrome or other unstable cardiopulmonary conditions [22,23,24,25]. After UEA injection, patients should stay for at least 15 min in the waiting area of the echo laboratory. Patients should be instructed to report to the staff when they feel unwell and/or have complaints about itching, breathing problems, or swallowing.

The three approved UEAs have not been tested in pregnant and lactating women. Women of childbearing age should be asked about pregnancy and/or whether they are breastfeeding.

There are only a few absolute contraindications [Table 3].

UEAs should not be administered in patients with a history of hypersensitivity to one of the components, such as polyethylene glycol (PEG) in SonoVue^®^ and Definity^®^ [35]. Optison^®^ and Definity^®^ are contraindicated in patients with perflutren hypersensitivity. Moreover, Optison^®^ is contraindicated in patients who have an allergy to blood products or albumin [24]. SonoVue^®^ is contraindicated in patients with known hypersensitivity to sulfur hexafluoride. Definity/Optison^®^ can be cautiously administered in a patient with pulmonary hypertension if it is needed to enhance right or left ventricular visualization [24,25]. SonoVue^®^ should not be administered in patients with severe pulmonary hypertension (>90 mmHg), known right-to-left shunts, and acute respiratory distress syndrome (ARDS) [23]. UEAs can be used in patients with a patent foramen ovale (PFO) with small degrees of right-to-left shunts [13]. Because of recent reports of pain-related adverse events, high doses of Definity should be avoided in patients with sickle cell anemia, although it is not yet listed in the package leaflets [36,37].

### 2.2. Informed Consent

Local recommendations should be followed. In some countries, it is requested that the patients sign a consent form after being informed about the need for an echocardiogram with UEA and the risks associated with the UEA. The physician or sonographer should ask about allergies to UEAs and components of UEAs and should mention the low risk of minor allergic reactions and the very rare risk of severe allergic or pseudoallergic reactions [38,39]. Because of their size, microbubbles have the tendency to interact with the innate immune system via circulating monocytes and other types of macrophages. Furthermore, they can interact with immunoglobulins like IgG and IgM and complement proteins by absorbing these proteins, generating a protein corona. Both mechanisms can contribute to the generation of anaphylatoxins like C5a and cause a complement activation-related pseudoallergy (CARPA) [40].

### 2.3. Safety of CEUS/How to Prepare for Adverse Events

In the early years after approval of the UEAs, rare fatal events in temporal relation with the administration of UEAs caused a temporary suspension for clinical use of SonoVue issued by EMEA and black box warnings for perflutren-containing UEAs by the FDA in 2007 [41]. This triggered several large studies on the safety of UEAs, which showed a very low risk of serious adverse events (severe allergic or pseudoallergic reactions) in about 1 in 10,000 examinations [13]. An echocardiography laboratory performing studies with UEAs should have all the drugs needed for allergic, anaphylactic, and cardiovascular emergencies and equipment for resuscitation, including all materials for intubation, endotracheal suction, ventilation, and defibrillation. Injection of UEAs should only be performed in the presence of staff trained in basic life support. Prompt treatment of adverse events depends on proper IV access. Therefore, UEA injection should only be performed after the correct position of the IV line has been confirmed with a bolus injection of 10–20 mL saline.

Alertness for and immediate recognition of pseudo anaphylaxis and the early use of epinephrine are essential for treatment and treatment rules follow general recommendations and guidelines [42]. Administration of H1 and/or H2 antihistamines and corticosteroids should be considered as adjunctive therapy and not as a substitute for first-line treatment with epinephrine. Oxygen should be considered for all patients experiencing anaphylaxis, regardless of their respiratory status. If bronchospasm is a component of anaphylaxis, an inhalable beta-agonist should be applied [42]. Mild adverse events such as headache, nausea, chest pain, feeling hot/flushing, and back pain (mainly with Definity^®^) usually resolve spontaneously, but may require symptomatic treatment.

### 2.4. Application of UEAs

A secure venous access, for example, with a peripheral venous line, is required. UEAs must be injected intravenously, usually via a cubital or forearm vein, preferably on the right arm. This provides a good flow to the heart when the patients are in the left decubitus position for transthoracic echocardiography. Furthermore, 20 G cannulas or larger are recommended as microbubbles may be degraded by a small-caliber intravenous cannula or tubing. **The intra-arterial administration of UEAs is NOT approved.** However, selective injections of UEA into a septal branch of the left anterior descending artery have been performed to delineate the perfusion territory of the septal branch before ablation in patients with hypertrophic cardiomyopathy [43].

#### 2.4.1. Bolus Injection

**Figure 1:**
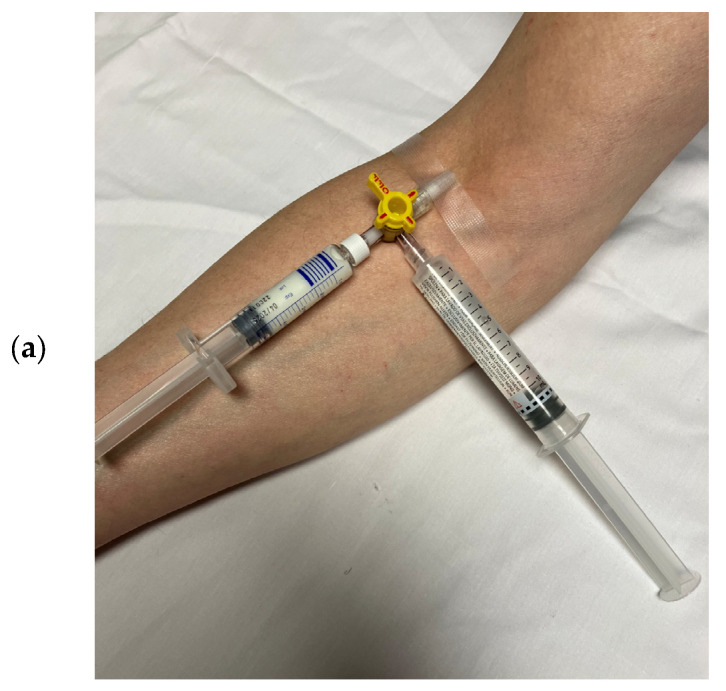

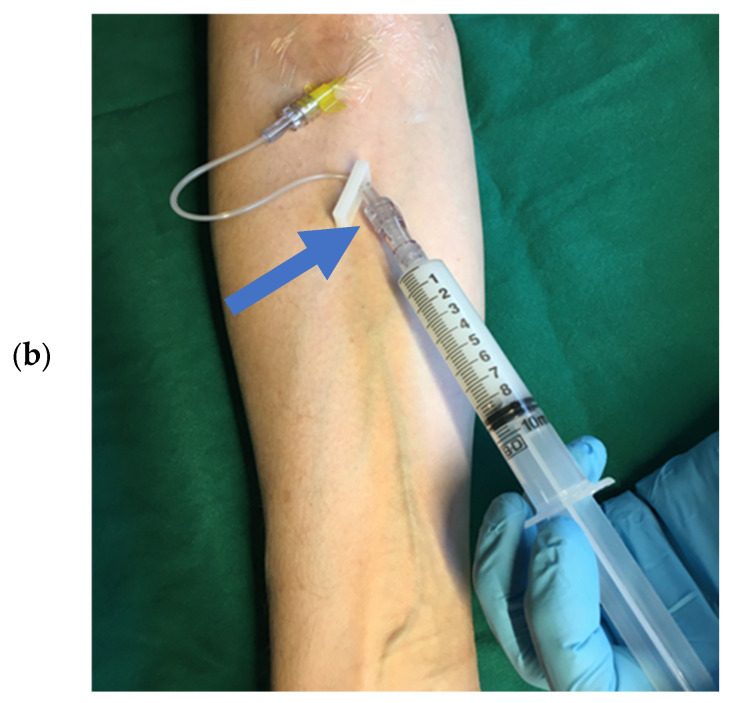
(**a**) Injection of UEAs with a flush via a three-way stop cock. For re-agitation, the syringe with the diluted UEA must be removed from the stop cock and tilted several times. (**b**) Injection of UEAs without a flush via a short extension line that connects the IV line with the 10 mL syringe, as well as the microclave, clamp, and rotating luer. For re-agitation of the diluted UEA, the echocardiographer picks the syringe at the luer connector (arrow) and tilts the syringe forward and backward. This way, there is less risk of pulling the IV, and the system remains closed.

Slow bolus injection is adequate for most indications. Pushing the UEA bolus with an additional flush has been recommended in the package leaflets of the manufacturers and recommendations/guidelines of cardiac imaging societies [Figure 1].

The UEA and the saline flush (0.9% NaCl solution) are injected via a three-way stop cock. The bolus [Table 2] followed by a 5–10 mL saline flush should be injected slowly (3–5 mL saline flush over a period of at least 5 s) [17]. This helps to reduce the initial attenuation effects of deeper parts of the LV. Using the recommended diluted solutions of Definity^®^/Luminity^®^ or SonoVue^®^/Lumason^®^, bolus injections can also be performed without flushing. A “closed” injection can be used with a short extension line that connects the IV line with the 10 mL syringe, as well as the microclave, clamp, and rotating luer. This setting allows the sonographer to perform the study without a nurse. Institutional review and agreement before implementation are required. Sonographers who perform injections of UEAs should have received appropriate training for inserting IVs, preparing/administering UEAs, and detecting adverse events [44,45]. The sonographer can move the syringe before injections to agitate the UEA by rotating and tilting the syringe a few seconds before injection. The recommendations for supervision by physicians or advanced practice professionals have been published in a recent multi-societal expert consensus statement on the safe administration of ultrasound contrast agents [46].

#### 2.4.2. Continuous Infusion via a Peripheral Line

**Figure 2:**
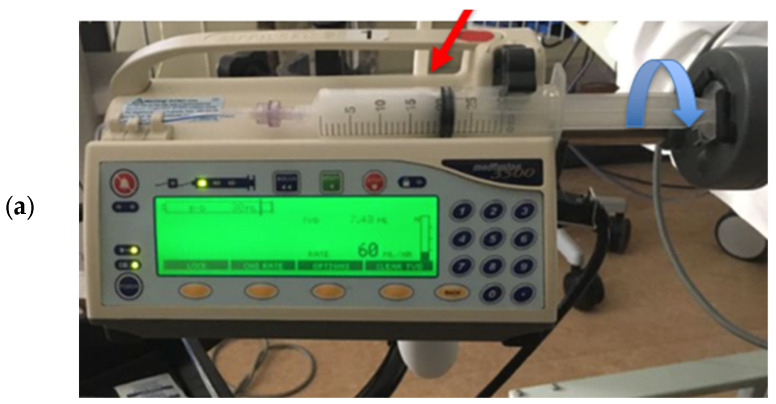

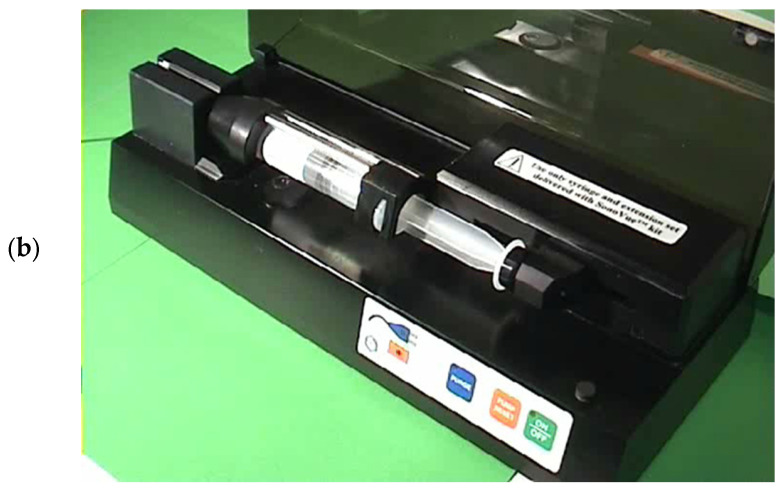
Infusion of UEA with infusion pump (**a**), syringe with diluted UEA (red arrow) can be manually rotated up to 180 degrees forward and backward every 3–5 min (blue arrow). For SonoVue a rotating pump (**b**) has been developed, in which the syringe is continuously rotated (**b**).

The infusion of UEAs is recommended for the assessment of myocardial perfusion. It takes at least 20 s until a steady state of concentration of the UEA in the heart is achieved. Table 2 shows the initial infusion rates, which may be adjusted to provide optimal LV opacification. Microbubbles float in solutions and separate into the higher parts of syringes or infusion bags. This can be mitigated by manual squeezing/movement of the bags or rotating the syringes in the infusion pump [Figure 2]. A specific pump for SonoVue^®^ (Vueject^®^) continuously oscillates and agitates the suspension and provides homogeneous distribution of the microbubbles in the solution. These pumps have been used in many echocardiography laboratories but are currently not available [Figure 2]. An updated version of the VueJect pump compliant with the new medical device regulations is in development.

#### 2.4.3. Bolus Injection/Infusion via a Central Line

UEAs can be injected or infused via a central line. However, the contrast effect of the recommended bolus or infusion rates can be stronger compared to the effects after injection into a cubital or forearm vein. It should be noted that resistance and shear stress increase with the length of the infusion line. Therefore, bolus injections should be performed slowly (<1 mL diluted UEA/min). No experience with injections into a Port catheter has been reported.

#### 2.4.4. Multiple Injection/Infusions of UEA Boli or Restarting UEA Infusion

Multiple injections/infusions are possible if the maximum dosage is not exceeded [Table 2]. Before a second injection is performed, the patients should be asked whether they experienced any discomfort. Also, the UEA must be re-agitated before it is injected/infused again. The UEA should be administered immediately after reconstitution.

#### 2.4.5. UEA Application in Patients with LVAD and ECMO Devices

Left ventricular assist devices (LVADs): The safety and diagnostic value of using UEAs have been shown in previous studies [47,48]. UEAs were tolerated well and led to improved visualization of thrombi and LV borders compared to 2D echocardiography without UEAs. The same UEA dosages as recommended for the assessment of LV function and thrombi in patients without LVAD can be used.

Extracorporeal membrane oxygenation (ECMO): Microbubbles may interfere with the bubble detector in the ECMO device which can lead to its malfunction. This can be addressed by pausing the bubble detector prior to the intravenous injection of the UEAs. Protocols on how to manage the ECMO device before and during the injection of UEAs have been published [49,50,51,52].

### 2.5. Perspectives of New UEAs

There is ongoing research on advancing microbubbles, including, for example, producing more uniform microbubbles, special microbubbles for local drug delivery, or microbubbles with ligands for molecular imaging [53]. However, it is unlikely that new agents with microbubbles will be available for clinical application within the coming 5 years.

### 2.6. What Should We Know About Contrast Phases?

After intravenous injection of the UEA, opacification of the RA and RV is observed within 5–10 s, depending on cardiac output. LA and LV opacification usually come after another 3–5 cardiac cycles (the 3–5 beat rule has been used to distinguish intracardiac from transpulmonary shunts using agitated saline) [54]. However, it may be shorter in high-cardiac-output scenarios. Within the next two cycles, epicardial coronary flow is clearly enhanced on color and the Pulsed-Wave Doppler, and, finally, about five cardiac cycles after LV opacification, the UEA can also be detected in the myocardial tissue. The interval between LV opacification and myocardial tissue opacification becomes shorter with myocardial hyperemia. This change is the basis for assessing myocardial perfusion with stress echocardiography [27]. However, for the assessment of myocardial perfusion, the flash-replenishment method is applied during continuous infusion of the UEA. Myocardial replenishment of contrast is assessed after clearance of microbubbles in the myocardium by a short sequence of frames with increased acoustic power [27].

The sequence of opacification is not relevant for the approved indications in echocardiography. A constant concentration of microbubbles in the blood rather than an up-and-down contrast in the cardiac chambers is better achieved with a continuous infusion of UEAs rather than repeated boluses. When bolus injection is performed, a slow bolus with diluted UEAs is preferable [Table 2]. When using contrast-specific imaging modalities, a high fraction of UEA microbubbles recirculate for some time, and it is usually possible to acquire several cardiac cycles with a similar contrast effect after the initial intense peak opacification of the cardiac chambers.

## 3. Imaging Modalities for UEAs

Contrast-specific imaging modalities should be used when UEAs are injected [Table 4] [8,13,14,15,16,17].

Contrast-specific imaging modalities enhance the signals from microbubbles and reduce the destruction of microbubbles in the sound field by applying a reduced transmit power and a specific pulsing scheme. On most echo machines, the same transducer is used for 2D echocardiography with and without UEAs. Standard 2D transducers often provide better contrast images compared to matrix transducers for both 2D and 3D imaging. The manufacturers of echocardiography scanners provide presets for low- and intermediate-MI contrast-specific imaging. The application specialists of the UEA manufacturers can help to adjust the preset for their UEA. The low-mechanical-index (MI) methods are particularly useful, as they provide simultaneous assessments of wall motion and myocardial perfusion and require fewer contrast agents compared to methods using higher MIs. For the optimal assessment of LV structure and LV thrombi, the additional use of intermediate-MI imaging is recommended. The low-MI contrast preset should be the default preset for LV studies with UEAs.

### Perspectives of Imaging Methods and Analysis Tools

While the development of new agents usually takes a long time, imaging technology and analysis tools progress faster. Using AI-based techniques, view detection, automated LV cavity segmentation, and LV volume calculations, EF, as well as GLS, can be performed [55,56]. The excellent segmentation of the cardiac chambers on recordings with UEAs is expected to enhance automated analysis. However, automated analysis is only just beginning to be implemented in commercially available scanners, and reference values must still be established.

Three-dimensional echocardiography is possible with UEAs, but more user-friendly tools are required for wider clinical acceptance [57]. Currently, there is no AI-based analysis software for 3D echocardiography, and no reference values for LV volumes and EF have been established.

## 4. Fundamentals of Practical Application

### 4.1. Left Ventricular Function

#### 4.1.1. Background and Indications

Echocardiography is the first choice for the assessment of global and regional systolic LV function. When imaging with UEAs does not provide adequate recordings or UEAs are contraindicated, cardiac MRI should be considered, which is more expensive than echocardiography with UEAs. Alternatively, gated blood pool scintigraphy can be used for measurement of the EF. However, gated blood pool scintigraphy should be avoided in younger patients because of radiation exposure.

The challenge in the segmentation of the LV borders on non-contrast images is due to the trabeculation of the endocardial layers. Ideally, the tracing of the endocardial borders should be performed along the interface between the trabeculated and compact myocardial layer [58]. Without UEA, the measurements are often made along the trabeculations which are often poorly visualized. In addition, apical reverberations and lung artifacts blur the endocardial borders.

**Figure 3:**
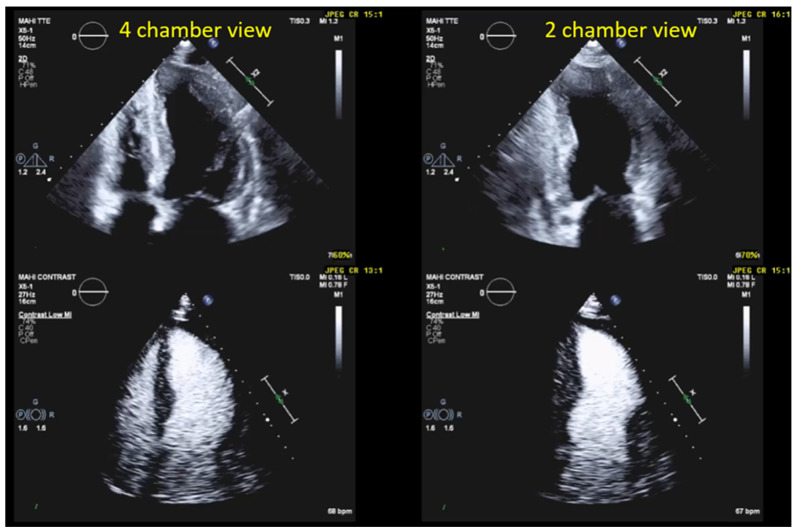
Improved delineation of endocardial left ventricular borders on recordings with UEA (power modulation, **bottom**). Unenhanced 4- and 2-chamber views (**top**) with poorly defined mid/apical lateral and anterior LV walls.

Contrast echocardiography is indicated when the display of the LV on non-contrast echocardiography may negatively affect the accuracy of LV volume and EF assessment or when regional LV-wall motion cannot reliably be assessed. According to the ASE (American Society of Echocardiography) and the EACVI (European Association of Cardiovascular Imaging) guidelines, this applies when the endocardium in two or more contiguous segments of the apical views is not clearly visualized and/or if accurate quantification is needed. The boundary of the trabeculated layer to the compact myocardium must be detectable for the quantification of LV volumes; however, this is often difficult on still frames, especially in apical LV segments, even when visual assessment is still possible while the loop is running [Figure 3]. For the clinical questions listed in Table 5, the UEAs should be considered in most patients.

#### 4.1.2. Advantages of Contrast-Enhanced Echocardiography

Two-dimensional echocardiography with UEAs outperforms conventional two-dimensional echocardiography regarding accuracy and interobserver variability, even in patients in whom image quality is deemed to be adequate on unenhanced images. The variability of EF measurements by echocardiography with UEAs is comparable to that of cardiac MRI. The evidence is summarized in the EACVI guidelines [16]. Echocardiography with UEAs provides a reliable assessment of systolic LV function in patients for whom MRI is not available or when patients are too ill to be transported to the MRI suite.

### 4.2. Preparation and Performance

#### 4.2.1. Technical Aspects and Settings

A preset for “low-MI” contrast imaging is recommended. The low-MI setting provides superior delineation and intensity of LV opacification compared to the intermediate-MI setting. Also, the low setting is more sensitive for the detection of UEAs and causes fewer artifacts in the nearfield. If there is no available “low-MI” setting, an “intermediate-MI” setting may be used. This is available on most echo machines as a preset (for example, contrast LVO). However, higher dosages may be necessary in the “intermediate-MI” mode. Presets of the contrast-specific imaging modalities can be uploaded by the support staff of the ultrasound equipment manufacturers. It should be noted that the MI (mechanical index) is not the same for the three contrast agents. When programming the preset, one should consider which UEA will be used. The sector depth should be set so that the LV and at least 1/3 of the left atrium below the mitral valve are displayed in the field of view. The focus should be set at the level of the mitral ring.

#### 4.2.2. Pre-Assessment and Primary Scan Plane

Contraindications should be checked for, and informed consent should be obtained. Then, the IV line (preferably on the right arm) is inserted or an existing IV is used. Table 2 shows the initial dosages that apply for all indications. Before turning on the contrast-specific imaging modality, the standard four- and two-chamber views are displayed and optimized using the standard 2D echocardiography (tissue harmonic mode).

#### 4.2.3. CEUS Procedure

Patients are scanned in the left decubitus position, like in non-contrast echocardiography. The recordings start with the four-chamber view, which is displayed in standard non-contrast 2D echocardiography. After injection of the UEA bolus, the standard non-contrast 2D mode should be maintained until the contrast appears in the RV, which usually takes 5–10 s. Then the contrast-specific mode is turned on. Because tissue signals are suppressed in the low-MI contrast mode, there are limited landmarks for the sonographer to keep the probe in the optimal position until UEA arrives in the left heart. In the low-MI modality, only the stronger signals come from the mitral ring and the pericardium. The examiner should not obsess over finding the optimal position of the probe. As soon as the contrast is visible in the left heart chambers, the sonographer can adjust the position of the probe to obtain unforeshortened views of the LV. After RV opacification, it takes about 5 s until the contrast agents reach the left heart chambers. After UEA bolus injection, there may be bright opacification of the apical LV cavity for several seconds with poor or missing contrast in the basal cavity (shadowing of the basal LV cavity) [Figure 3]. It can take several seconds until complete opacification of the LV cavity and the proximal LA is obtained. At that point, recordings can be obtained (see below). It is recommended to record loops with at least two cardiac cycles (sinus rhythm) or five cardiac cycles (atrial fibrillation) [Table 6].

### 4.3. Optimization of LV Opacification

**Figure 4:**
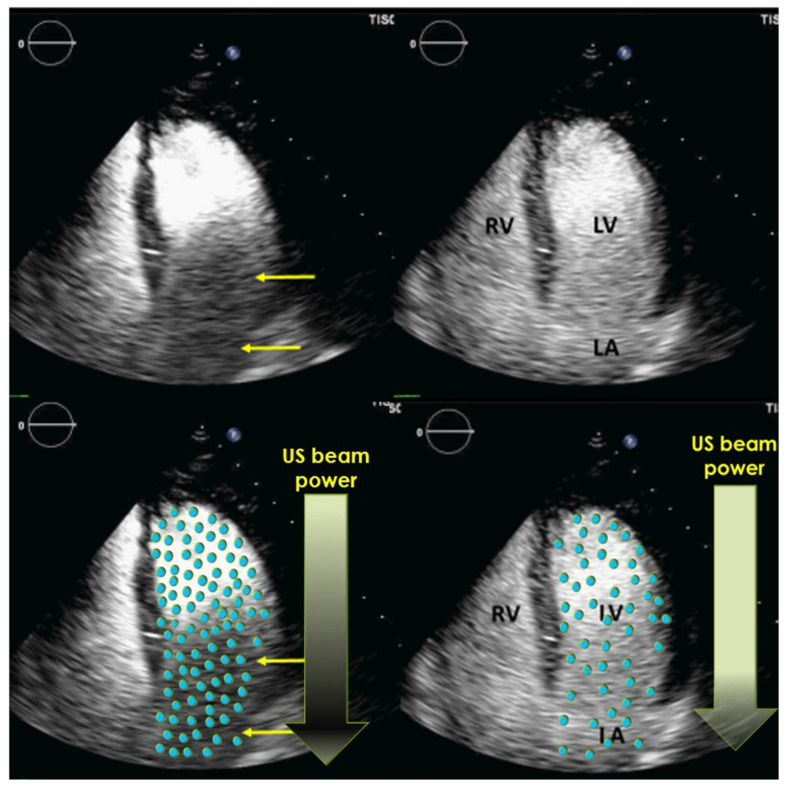
Contrast shadowing: 4-chamber view (left) due to high concentration of microbubbles in the LV early after bolus injection. The ultrasound is attenuated in the nearfield and does not reach the basal LV cavity. At greater depth the microbubbles do not resonate which causes poor LV opacification (yellow arrows). Notably, 20 s later and without any change in machine settings (right), there is a higher degree of dilution of the microbubbles, and there is also enough power from the ultrasound to resonate the microbubbles at a greater distance from the transducer. US: ultrasound.

**Figure 5:**
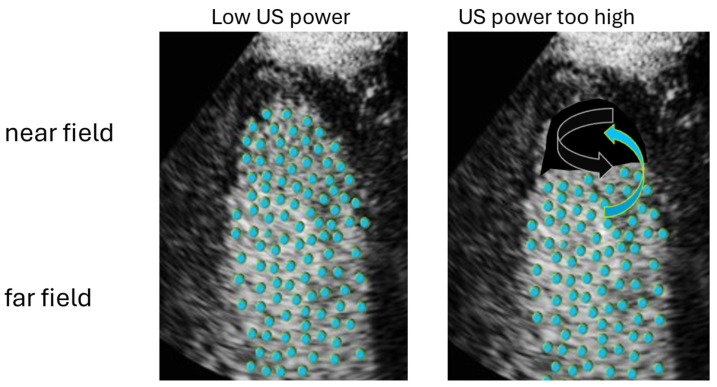
When the transmit power (MI) of the transducer is too high, the microbubbles in the nearfield are destroyed (black arrow). Blood with no bubbles is mixed with blood with intact microbubbles (blue arrow) coming through the mitral valve.

**Figure 6:**
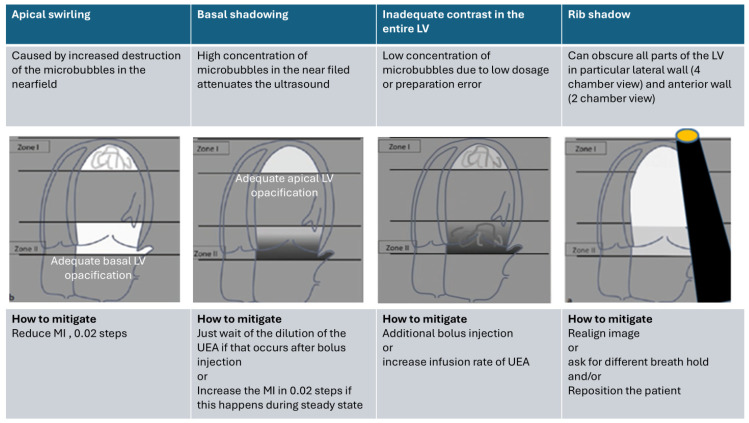
Optimization of LV opacification in apical views. Adjustments are made according to the intensity of completeness of the opacification in the apical and basal third of the LV (zones 1 and 3). MI: mechanical index.

For optimization, Figure 4, Figure 5 and Figure 6 show the typical patterns of artifacts and advise on how to mitigate the artifacts. For optimization, the apical and basal third of the LV cavity should be examined first. Recording with homogeneous LV contrast with no swirling or attenuation should be obtained. Farfield shadowing (attenuation) can be avoided by lowering the dosage (diluting) of UEAs and waiting after the bolus injection. Nearfield swirling is either caused by an increased destruction of microbubbles or an injection of a lower-than-ideal dose of UEAs (or too diluted); the blood without microbubbles near the apex is mixed with the intact microbubbles coming through the mitral valve. Nearfield swirling can be mitigated by reducing the transmit power or increasing the microbubbles. When nearfield artifacts are displayed, the transmit power (MI) should be reduced by steps of 0.02, and the LV opacification is assessed until there is a homogeneous LV opacification.

### 4.4. How to Avoid Foreshortened Imaging Planes

**Figure 7:**
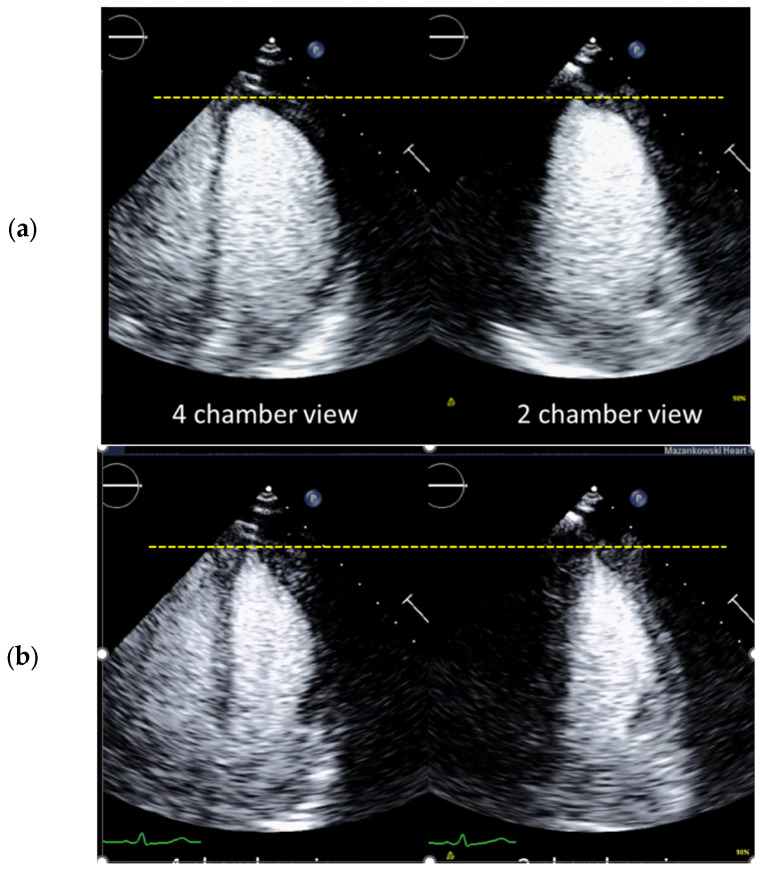
Triangular shape of LV apex in systole and no change in the apex position between diastole (**a**) and systole (**b**).

**Figure 8:**
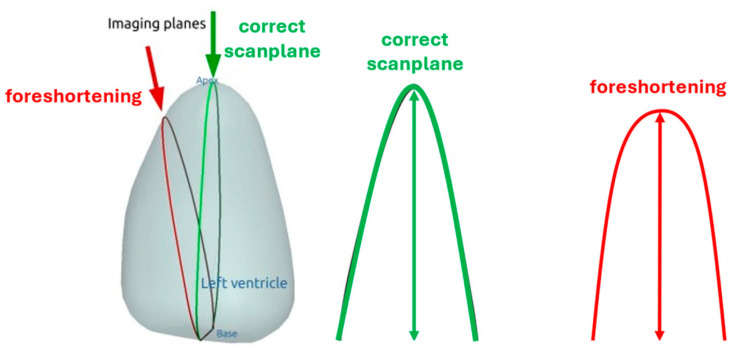
How apical foreshortening (red) affects LV length and the shape of the apex; modified from Smistad E. et al. [17,59].

For accurate measurements of LV volumes and ejection fractions, the imaging planes must intersect the apex and the middle of the mitral ring. The LV opacification displays the blood pool delineated by the compact myocardium and covers the trabeculations. This provides a much better visual assessment of the LV apex compared to the often-noisy display on unenhanced echocardiography. First, the imaging plane with the longest LV is searched for. There should be no change in the apex position between diastole and systole [Figure 7] except in patients with apical aneurysm in whom there may be a dyskinetic movement in systole. In patients with normal LV function, looking at the shape of the LV apex helps to avoid foreshortened imaging planes: The typical triangular shape is usually well displayed in systole. A rounded apex is suspicious for foreshortening. The display of focal apical thinning also indicates that the scan plane intersects the apex [Figure 8] [17,59] [Table 7].

### 4.5. Left Ventricular Volume and Ejection Fraction Measurements

The biplane Simpson method is recommended to measure LV end-diastolic and end-systolic volumes [Table 8] [60].

#### 4.5.1. Pitfalls

##### Selection of Foreshortened Loops

All recorded loops should be viewed before the evaluation. One should not simply select the first one that comes along for analysis. Table 7 shows the criteria for unforeshortened views.

##### Selection of False End-Diastolic and End-Systolic Frames

Slow scrolling through the loop using the arrow keys (instead of the mouse) facilitates the visual identification of the frame with the largest and smallest LV area. After tracing the borders of the LV cavity, toggling to the previous and following frames of the end-diastolic and end-systolic frame is useful to confirm the correct selection of the end-diastolic and end-systolic frames.

##### Starting Point for the LV Contour at the Mitral Valve Ring Is Incorrect

Oftentimes, the mitral valve ring is easier to recognize when scrolling through the loop than on still frames. When the mitral valve is not well visible, reviewers should watch for calcifications and strong echoes in the mitral valve ring. These are often better seen at the lateral ring than at the septal ring. The septal ring is difficult to recognize if a five-chamber view is recorded instead of the correct four-chamber view. The septal hinge point of the anterior mitral valve should then be assumed to be the same depth as the lateral mitral ring (this can be verified in the 2D registration without a contrast agent).

##### Wrong Contour/Papillary Muscle

**Figure 9:**
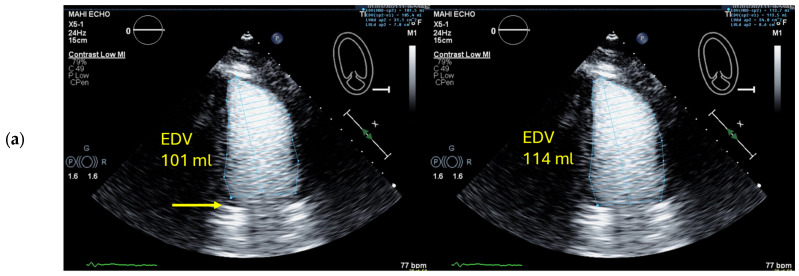

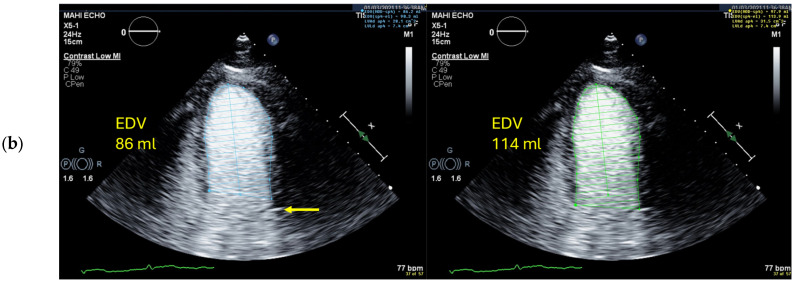
Identification of the bright echoes of the mitral ring to set the start and end points for tracing the LV cavity. The tracing should start just above the bright echoes (arrow), which are usually displayed on the anterior and inferior ring in the 2-chamber view (**a**). In the 4-chamber view, a bright echo is usually better seen at the lateral ring (**b**). It should be noted that underestimation occurs when the tracing does not start at the ring (left frames).

**Figure 10:**
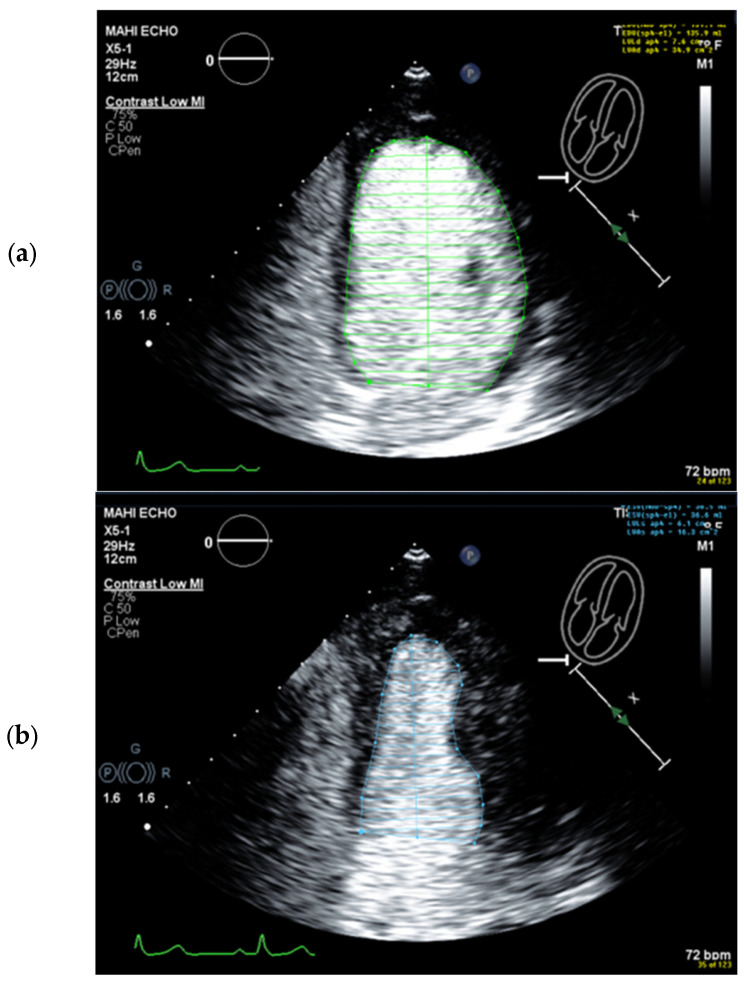

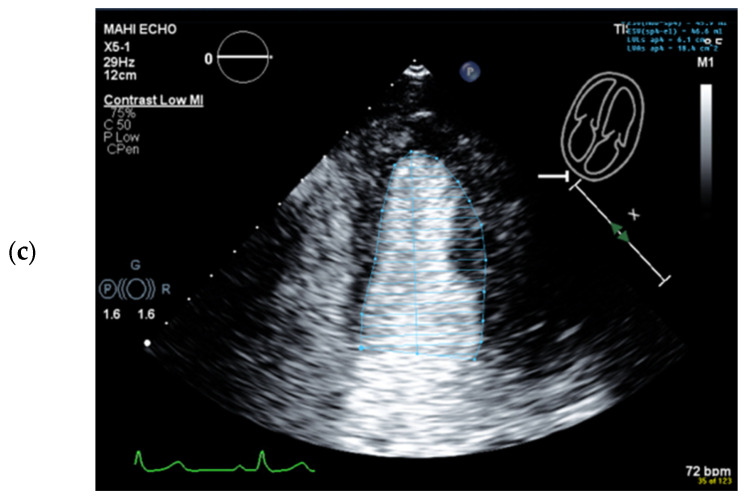
Problem with having the anterolateral papillary muscle displayed in the 4-chamber view: (**a**) End-diastolic frame with correct tracing of the LV borders (green). Part of the anterolateral papillary muscle is displayed; however, the lateral wall could be easily traced. (**b**) End-systolic frame, incorrect tracing (blue). There is less distance between the papillary muscle and the lateral wall, and the tracing follows the contour of the papillary muscle. (**c**) End-systolic frame, correct tracing (blue). After replaying the loop and moving frame by frame, the LV lateral border was adjusted.

**Figure 11:**
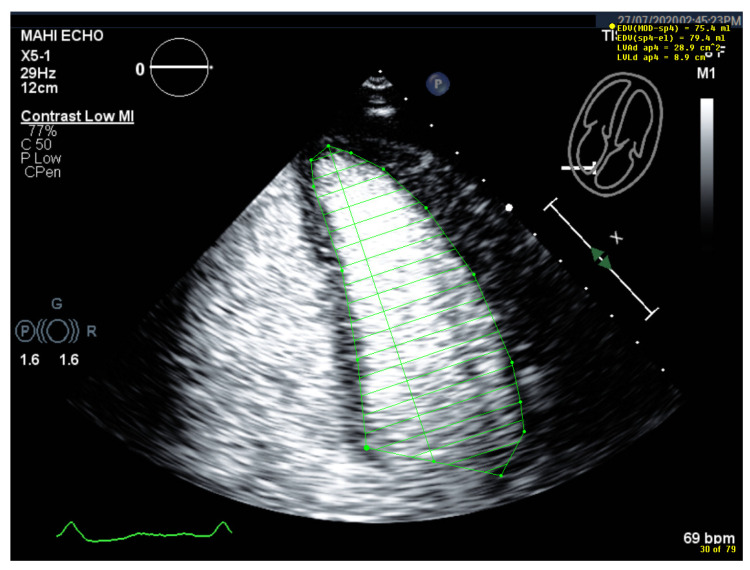
Incorrect starting point of LV tracing at the medial mitral ring. The tilted base of the tracing (green) should be noted. When the septal mitral ring is not clearly delineated, the starting point for tracing the LV cavity can be assumed at the same distance as the lateral mitral ring, which is usually easy to track. One can also review the non-contrast recordings.

With the “low-MI” contrast setting, there is also a contrast increase in the myocardium, especially in the infero-septum and inferior wall, which can impair the delineation of the LV cavity. An adequate delineation of myocardium from the LV cavity can often be achieved by reducing the brightness or gain using the post-processing tools on the echo machine or workstation [Figure 2]. If papillary muscles are displayed (i.e., in a two-chamber view), they should be counted toward the cavity. On the workstation, the amplification of the gain and brightness can sometimes show the opacification outside the papillary muscle, which allows contouring. Otherwise, the LV/myocardial border is interpolated [Figure 9, Figure 10 and Figure 11].

### 4.6. Interpretation and Reporting

#### 4.6.1. Global Systolic LV Function

The reference values for the global left ventricular ejection fraction (EF) are the same when using 2D echocardiography with and without UEA [Table 9].

According to the 2015 ASE/EACVI recommendations for chamber quantification, 52% is the lower level of normal for men, and it is 54% for women [58]. The classification as mild, moderate, or severely reduced LV function is not different in 2D echocardiography with UEA. However, the end-diastolic and end-systolic volumes measured on recordings with UEA are larger than those obtained without UEA. When tracing the borders of the LV blood pool, the entire volume inside the compact myocardial layer is determined. The UEA fills the gaps between the trabeculations up to the compact myocardium. Without UEA, this border is not well displayed and is usually estimated within the trabeculated layer. No reference values for LV volumes have been published by imaging societies. There is limited data from single-center studies on control patients with no risk factors and patients undergoing stress echocardiography, in whom normal volumes were measured in non-enhanced echocardiography, and low-MI contrast-specific imaging was used. LV volumes on recordings with UEA are about 20–30% larger than LV volumes in 2D echocardiograms without contrast, and interobserver variability is lower than in standard echocardiography [61].

#### 4.6.2. Regional (Segmental) Wall Motion

Visual assessment of segmental thickening and inward motion of the endocardium is performed by applying the same criteria as for non-contrast imaging. Because of the delineation of the interface between the compact and the trabeculated myocardium, the display of the movement of the myocardial segments toward the center of the LV is enhanced, which facilitates visual assessment. Normal myocardium thickens up to 50% in systole. Regional wall motion abnormalities on UEA recordings are classified in the same way as on non-contrast studies. Mid-cavity LV segments, often obscured by papillary muscles, are much more easily assessed for active myocardial thickening with UEAs.

#### 4.6.3. Left Ventricular Strain Analysis

GLS measurements are usually performed in addition to EF measurements. However, for reliable results, good image quality is required. If more than two segments in any one view are not adequately tracked, the calculation of GLS should be avoided [62]. The software on commercially available echocardiography systems has not been designed to measure global longitudinal strain (GLS) on recordings with UEAs. However, there have been studies using manual tracking of the LV to measure GLS. At present, there have been no major studies, and no normal values have been established.

### 4.7. Alternative Imaging Methods

**Figure 12:**
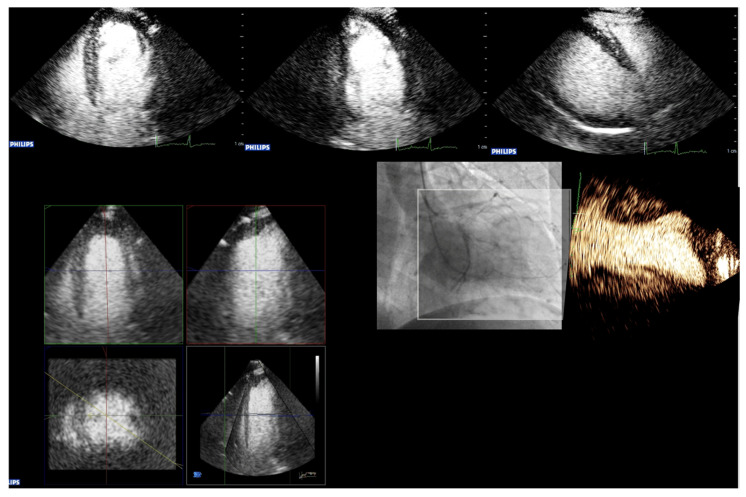
Three-dimensional echocardiography may be useful to intercept abnormalities in non-standard scan planes, as this anterolateral aneurysm would be fully missed if using only standard 4-, 2-, and 3-chamber views. The top row shows the standard apical views obtained by 2D contrast echocardiography. The quad screen on the left shows the standard reconstructed 2D planes from 3D contrast echocardiography. The anterolateral aneurysm is only displayed on a non-standard view (colorized frame mid-right).

Three-dimensional echocardiography studies have been performed with UEAs. However, due to limited evidence, no recommendation can be provided. Three-dimensional echocardiography can be helpful in identifying wall motion abnormalities that are not seen in standard 2D [Figure 12]. The reproducibility of EF measurements on 2D recordings with UEAs is significantly better than on non-contrast images. When UEAs are contraindicated, the method of choice for assessing the LV function is cardiac MRI. Gated blood pool scintigraphy is an alternative in older patients [63]. However, this modality is associated with radiation exposure.

## 5. Conclusions

Cardiac contrast-enhanced ultrasound is a patient-safe modality that often improves the precision of measurements and diagnostic conclusiveness compared to conventional 2D echocardiography. Although relatively easy to perform, a profound knowledge of indications and contraindications, specific imaging modalities, optimization procedures, and potential pitfalls is necessary to yield the potential added value that this technique harbors. Alongside these important features, this article elaborates practical application of CEUS for specific echocardiographic questions. Cardiac contrast-enhanced ultrasound is a valuable and safe imaging alternative for critically ill patients, especially for those with severely impaired kidney function. Standardized and repeated training of novices by experienced members of the echocardiography laboratory is crucial for quality assurance and control.

## Figures and Tables

**Table 1:** Properties of current commercially available UEAs for echocardiography.

	SonoVue^®^/Lumason^®^ Bracco Imaging, Milan, Italy [22,23]	Optison^®^ GE Healthcare, Marlborough, MA, USA [24]	Luminity^®^/Definity^®^ Lantheus Medical Imaging, North Billerica, MA, USA [25]
Gas	Sulfur hexafluoride, SF_6_	Perflutren, C_3_F_8_/octafluoropropan	Perflutren, C_3_F_8_, octafluoropropan
Shell	Phospholipid	Human albumin	Natural lipids (DPPC, DPPA), synthetic lipid MPEG 500 DPPE
Gas/mL	45 μg/mL	220 +/− 110 μg/mL	150 μg/mL
Mean size of microbubbles	1.5–2.5 μm	3–4.5 μm	1.1–3.3 μm
Bubble concentration	1.5–5.6 × 10^8^/mL	5–8 × 10^8^/mL	Up to 1.2 × 10^10^/mL
Storage	Room temperature	Refrigerator, 2°–8 °C	Refrigerator, 2°–8 °C Room temperature for Definity^®^RT *
Withdrawal from the vial, preparation #	No venting needle required; a special spike is provided in the package When a UEA is left in the vial for later injection, close the spike	Minimize positive and negative pressure by venting the vial and slowly withdrawing the UEA from the vial Remove the venting needle after withdrawal of the UEA	Minimize positive and negative pressure by venting the vial and slowly withdrawing the UEA from the vial Remove the venting needle after withdrawal of the UEA

# Follow the instructions of the manufacturers; * Definity RT (room temperature) is a special preparation of Definity^®^ which can be stored at room temperature. Specific advice for the preparation and administration of each UEA can be found on the ICUS (International Contrast Ultrasound Society) website [14]: http://icus-society.org/resources/product-labels/, accessed 1 July 2025.

**Table 2:** UEA dosages for 2D echocardiography. For the enhancement of PW, CW, and color, Doppler signals lower dosages are required. Doppler recordings can be performed in the wash-out phase after a bolus injection or after interrupting the UEA infusion.

	SonoVue^®^/Lumason^®^ [22,23]	Optison^®^ [24]	Luminity^®^/Definity^®^ [25]
*Bolus injection* package inserts *	*2 mL undiluted suspension followed by 5 mL 0.9% sodium chloride flush*	*0.5 mL followed by slow flush of 0.9% sodium chloride injection or 5% dextrose injection*	*10 microL/kg intravenously over 30 s to 60 s followed by a 10 mL flush of 0.9% sodium chloride injection;*
**Bolus injection** for current state-of-the-art scanner **	0.5 mL undiluted suspension or 1 mL diluted suspension (1 vial + 5 mL 0.9% saline flush) #	0.3–0.5 mL with slow 5- to 10-mL saline flush (15)	1–2 mL of diluted Definity^®^ (1.3 mL activated Definity with 8.7 mL of preservative-free saline in a 10 mL syringe) 1 mL of the diluted suspension (0.5 mL in 9.5 mL 0.9% NaCl) #
Initial infusion rate **	0.7 mL/min of undiluted suspension or 1.4 mL/min of diluted suspension (1:1 with 0.9% NaCl) #	1–4 mL/min of diluted suspension (3 mL in 30 mL 0.9% NaCl)	4 mL/min of diluted suspension (1 vial, 1.3 mL in 50 mL 0.9% NaCl -saline bag) #; not to exceed 10 mL/min 1 mL/min of diluted suspension (1 vial in 30 mL 0.9% NaCl syringe) #
Maximum dosage	Max. 2 mL/undiluted dose; a second dosage may be administered.	The maximum total dose should not exceed 5.0 mL in any 10 min period. The maximum total dose should not exceed 8.7 mL in any one patient study.	Two bolus doses of undiluted Definity (5 to 30 min apart, depending on EU, CA, or US) or one single infusion.

* The dosages in the package leaflets are often too high when using state-of-the-art scanners for other indications, like, for example, liver lesions. ** Adjusted according to LV opacification. **# Dilution of SonoVue^®^ and Definity- should be performed using 0.9% NaCl without preservatives!**

**Table 3:** Absolute contraindications for UEAs.

**All UEAs**	Pregnant and lactating women have not been included in studies for clinical license.
SonoVue^®^ *	Previous hypersensitivity reactions to SonoVue^®^/Lumason^®^ components of UEAs such as polyethylene glycol (PEG, macrogol), or PEG-containing products such as certain bowel preparations for colonoscopy or laxatives. Severe pulmonary hypertension (>90 mmHg). Acute respiratory distress syndrome (ARDS). Known right-to-left shunt. In combination with dobutamine in patients with conditions suggesting cardiovascular instability where dobutamine is contraindicated.
**Luminity^®^/Definity^®^**	Previous hypersensitivity reactions to these UEAs, components of UEAs such as polyethylene glycol (PEG, macrogol), or PEG-containing products such as certain bowel preparations for colonoscopy or laxatives. **CAVEAT:** Patients with sickle cell disease are particularly susceptible to adverse nociceptive events at high doses.
**Optison^®^**	Previous hypersensitivity reaction to Optison^®^. Allergy to blood products or albumin.

* In the US, SonoVue^®^ is licensed as Lumason^®^ with only contraindication ‘Hypersensitivity to sulfur hexafluoride lipid microbubbles or its components, such as polyethylene glycol (PEG)’.

**Table 4:** Contrast-specific imaging modalities for echocardiography with UEAs.

	Low-MI Method *	Intermediate-MI Method *
Mechanical Index (MI)	<0.2 *	0.2–0.5
Applications	First-line method for all LV indications	May be used in addition for LV thrombi; excessive LV trabeculation
Strengths	High sensitivity for detecting UEA Myocardial tissue signals minimized Display of contrast in myocardial vessels for assessment of perfusion	Higher spatial resolution
Limitations	Spatial resolution is worse than in the intermediate-MI method	More microbubble destruction; apical swirling is more likely Myocardial tissue signals, which limit delineation of cavity/myocardium

* The modalities may be named differently depending on the manufacturer. The low-MI method can be found as MCE (myocardial contrast echocardiography), and the intermediate-MI method as the LVO (LV opacification) method. It should be noted that the low-MI method is the first choice not just for the assessment of myocardial perfusion but for other indications as well. For the low- and intermediate-MI modalities, the manufacturers of echocardiography machines provide presets for the different UEAs. These presets set the mechanical indices, the compression, and the frame rate. However, sector depth/width, focus, and gain often need to be adjusted according to the clinical question.

**Table 5:** Assessment of systolic LV function—indications for UEAs according to EACVI/ASE/BSE guidelines. The indications for echocardiography with UEAs to assess myocardial disease and cardiac masses can be found in part 2.

When two or more contiguous segments are not clearly visualized. When the management of the patient depends on whether there are regional wall motion abnormalities or not, and when segments are not clearly visualized. Irrespective of image quality, when clinical monitoring depends on accurate measurements of EF, such as the assessment of cardiotoxicity due to chemotherapy in cancer patients or in patients considered for treatment with ICD or CRT devices.

**Table 6:** Quality criteria for LV opacification—assessment of global and regional LV function.

The entire LV is opacified, including 2 cm of the proximal LA (which facilitates identification of the mitral ring as landmarks for measurements of LV volumes). No major swirling in the apical cavity. No attenuation of the basal cavity. No rib shadowing (such as a straight LV border of the lateral wall in the four-chamber view or the anterior wall in the two-chamber view) is suspicious. The enhancement of the myocardium should be lower than that of the cavity (which facilitates segmentation of the myocardium from the blood pool). No foreshortening of apical planes.

**Table 7:** Quality criteria for identification of unforeshortened apical LV imaging planes.

LV length—try to find the recording with the longest LV. The apex does not move (except for apical aneurysms). Triangular shape of LV apex (when LV function is normal). Focal apical myocardial thinning may be displayed.

**Table 8:** Measurement of LV volumes and ejection fractions using the biplane Simpson method in 4- and 2-chamber views, step by step.

**1**	**Select unforeshortened loops** **avoid 4-chamber views, which** **include anterolateral papillary** **muscles**	When several loops are available, select the one with the longest long axis which impairs tracing of the lateral LV wall
**2**	**Select the end-diastolic frame**	The first frame after mitral valve closure or the largest LV cavity
**3**	**Trace the LV border: end diastole**	Starting on the septal mitral ring (4-chamber view) and inferior mitral ring (2-chamber view), following the dark/bright interface until the lateral/anterior mitral ring The start and end points of the contour at the mitral ring should be connected by a straight line *TIP* *The mitral leaflets may be obscured by the UEA. Then look for the bright signals of the mitral ring, which are more clearly delineated and follow the entire loop rather than only in the end-diastolic frame.*
**4**	**Select the end-systolic frame**	Smallest cavity—the mitral valve closure may be obscured by the UEA! *TIP: When scrolling through the systolic frames of the loop, look for the frame when the cavity becomes larger. The frame before it is the end-systolic frame.*
**5**	**Trace the LV border: end systole**	Starting on the septal mitral ring (4-chamber view) and inferior mitral ring (2-chamber view), following the dark/bright interface until the lateral/anterior mitral ring The start and end points of the contour at the mitral ring should be connected with a straight line
**6**	**Check the LV length (distance between the middle of the line connecting the mitral ring and the apex)**	When the difference in diastolic LV length between 4- and 2-chamber views is <0.5 cm, no major foreshortening can be assumed When the difference is >5 mm, the recording of the view with the shorter LV length is probably not optimal. The other recordings of this view should be reviewed to find the one with the longest LV [59].

**Table 9:** Two-dimensional echocardiography with UEAs for assessing LV function.

**Imaging method**	Low-MI contrast-specific imaging. Additional intermediate-MI imaging in case of apical aneurysm for the assessment of thrombi.
**Sector depth/width**	Adjust to display the entire LV and 1/3 of LA.
**Focus**	Midventricular or basal.
**Compression**	Adjust to enhance any signals from contrast agents; a high dynamic range is not necessary. The preset of the manufacturers is usually adequate.
**Gain**	Adjust to enhance the delineation of the LV. Often, a reduction in the gain is required to reduce the myocardial contrast signals.
**Automated segmentation and volume calculation**	Currently introduced by one manufacturer.
**Imaging planes**	Standard apical views and apical sweeps to assess the LV cavity and search for thrombi. The aneurysm may only be displayed in non-standard views [Figure 12].
**Contrast application**	Bolus injections.
**Typical findings**	LV cavity is well delineated during the entire cardiac cycle.
**Alternative imaging**	MRI when there are contraindications to UEAs or inadequate recordings despite the use of UEAs. Gated blood pool scintigraphy and CT in patients with contraindications for MRIs and UEAs.

## Data Availability

The data presented in this study are available upon request from the corresponding author. Some of the data originates from articles of other authors (see references). The data are not publicly accessible, as the personal rights of the patients involved must be respected.
